# Cryopreservation of tissues and organs: present, bottlenecks, and future

**DOI:** 10.3389/fvets.2023.1201794

**Published:** 2023-05-25

**Authors:** Jiangming Chen, Xiangjian Liu, Yuying Hu, Xiaoxiao Chen, Songwen Tan

**Affiliations:** Xiangya School of Pharmaceutical Sciences, Central South University, Changsha, Hunan, China

**Keywords:** cryopreservation, tissue and organ, cryoprotectants, vitrification, organ transplantation

## Abstract

Tissue and organ transplantation continues to be an effective measure for saving the lives of certain critically ill patients. The organ preservation methods that are commonly utilized in clinical practice are presently only capable of achieving short-term storage, which is insufficient for meeting the demand for organ transplantation. Ultra-low temperature storage techniques have garnered significant attention due to their capacity for achieving long-term, high-quality preservation of tissues and organs. However, the experience of cryopreserving cells cannot be readily extrapolated to the cryopreservation of complex tissues and organs, and the latter still confronts numerous challenges in its clinical application. This article summarizes the current research progress in the cryogenic preservation of tissues and organs, discusses the limitations of existing studies and the main obstacles facing the cryopreservation of complex tissues and organs, and finally introduces potential directions for future research efforts.

## 1. Introduction

Currently, the transplantation of human tissues and organs is recognized as an effective treatment for certain serious diseases. However, according to conservative estimates by the World Health Organization, <10% of the global demand for organ transplants is met, and the COVID-19 pandemic has exacerbated the supply-demand imbalance for human organs (1–3). One significant reason for the shortage of organs is the short safe storage time of donated organs, typically 4–36 h, causing a large number of healthy organs being discarded after exceeding the preservation time (4, 5). At present, the main methods used clinically to extend organ preservation time are static cold storage (SCS) (6) and machine perfusion (MP) (7–9). The preservation of most organs involves immersion in a preservation solution at a temperature range of 0–4°C, which reduces organ metabolism (10). For instance, SCS can typically preserve the heart for ~4 h and the liver for 12 h (11, 12). But metabolic activity does not completely cease during SCS, and the accumulation of toxic metabolites over time can cause organ damage. Recently, machine perfusion has attracted great interest due to its ability to provide oxygen and nutrients for cellular metabolism and remove toxic metabolites that can lead to ROS production, thereby reducing ischemia-reperfusion injury (9, 13). For short-term preservation, methods such as SCS and MP have made some progress compared to the previous ones, but still do not meet the requirements for long-term storage of tissues and organs.

According to the Arrhenius equation, the rate of cellular metabolism slows down with decreasing temperature. It is generally believed that tissues and organs can be stored for extended periods at temperatures below −130°C. Cryopreservation involves maintaining the physiological function of cells, tissues, and organs at ultra-low temperatures (−80°C or −196°C), at which point cellular activities nearly cease. Cryopreservation can effectively halt biological time and overcome the limitations of existing low-temperature storage technologies, making it a powerful tool for achieving long-term storage of tissues and organs (14–16).

The implementation of cryopreservation is not without challenges. For cells, the primary cause of damage during cryoprotective processes is the formation of ice crystals (17, 18). During slow cooling, ice crystals form outside the cells and gradually grow, causing mechanical damage to cells. Simultaneously, water inside the cells will extrude, leading to an increase in intracellular solute concentration and causing osmotic injury. While rapid cooling results in the dominant form of intracellular ice due to insufficient time for the water inside the cells to escape. This leads to mechanical damage to cell organelles, nuclei, and other structures (19, 20). Additionally, during the rewarming process, the recrystallization process can generate large ice crystals, which causes even more severe mechanical damage to the cells (18). So various cryoprotective agents (CPAs), such as glycerol (21), DMSO (22), and trehalose (23), have been used to reduce the damage caused by ice crystals.

**Figure 1:**
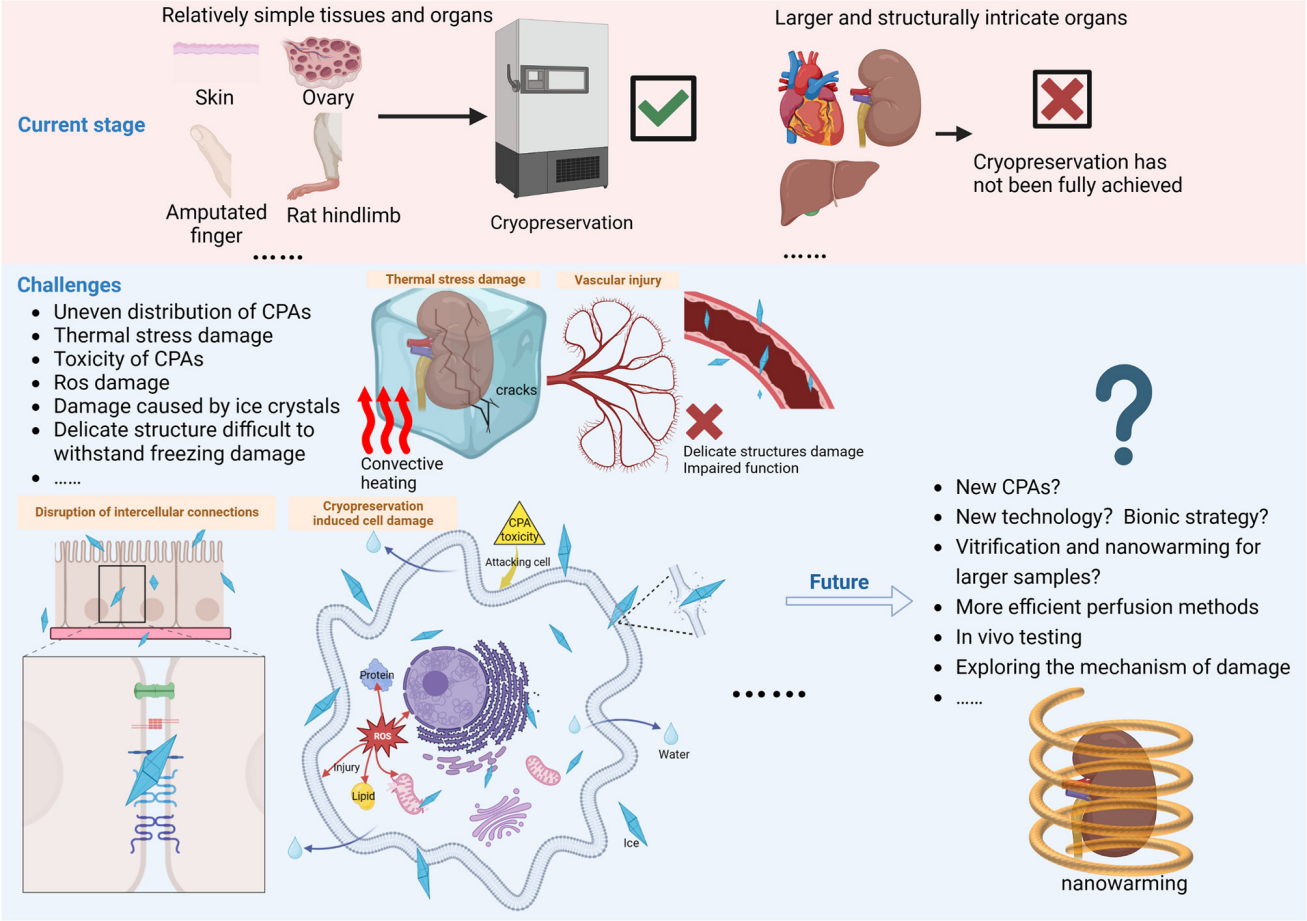
A few tissues and organs have been cryopreserved and transplanted, but cryopreservation of larger and more complex tissues and organs has not yet achieved clinical application. It faces challenges posed by inhomogeneous heat and mass transfer, thermal stress, CPA toxicity, reactive oxygen species, and ice crystals, requiring comprehensive protection measures in terms of macroscopic volume, fine structure, cellular connectivity, and individual cells. Vitrification and nanowarming has made a breakthrough in the volume of cryopreserved biological samples, applying it to the cryopreservation of larger tissues and organs and performing *in vivo* experiments to evaluate the recovery effect is the direction of future efforts. In addition, the mechanism of damage during cryopreservation needs to be further elucidated in order to explore new CPAs and new technical strategies to accelerate the development of cryopreservation technology. Created with BioRender.com.

At present, cryopreservation has been applied in clinical settings for certain types of cells, but the damage factors and mechanisms during cryopreservation of larger tissues and organs are more complex (24). In the cryopreservation of tissues and organs, it is not only crucial to consider the survival of individual cells, but also the preservation of the ability of cell-to-cell interactions, which makes it challenging for most complex tissues and organs to meet the clinical application requirements after cryopreservation (8, 25). This paper provides an overview of the current state of research on the cryopreservation of tissues and organs, covering commonly used techniques and their potential for development. We also describe the challenges encountered in cryopreserving complex tissues and organs, like thermal stress damage resulting from complicated heat and mass transfer, and discuss possible future directions (as shown in Figure 1).

## 2. Current status of tissue and organ cryopreservation

The history of cryopreservation of mammalian organs may be traced back to Gonzales and Luyet's attempt to freeze chick embryo hearts by vitrification in 1950 (26). Over the decades, people have never stopped exploring and have tried to cryopreserve a variety of tissues and organs (as shown in Table 1), and the most commonly used cryopreservation techniques include conventional slow freezing, vitrification, and directional freezing (14). A comparison of their strengths and weaknesses is in the Supplementary Table S1. At present, the technically mature programmable slow freezing method has established standard protocols for cryopreserving various types of cells (such as red blood cells and reproductive cells) (27, 28) and has been developed for clinical cryopreservation of some tissues [such as ovaries (29) and skin (30)]. Conventional slow freezing protocols typically employ a cooling rate of 1°C/min and 1.5 M CPA for cell freezing, the whole process allowing relatively free formation and growth of ice crystals, which is usually fatal for cryopreservation of large-volume tissues and organs.

**Table 1:** Summary of research on cryopreservation of some tissues and organs.

**Cryopreservation objects**	**CPAs**	**Main methods**	**Outcome/conclusion**	**References**
Rat heart	1.4 M DMSO	Slowly freeze to −30°C	Hearts can be reanimated after cooling	(104)
Rat hindlimb	DMSO, fetal bovine serum, trehalose/DMSO, ethylene glycol, trehalose	Directional freezing to −80°C/vitrification	Vessel integrity, color, and pliability were indistinguishable from the fresh recipient vessels; after transplantation all limbs survived until planned extraction	(35)
Rat hindlimb	FBS, DMSO, sucrose RPMI1640 medium	Programmatically cool to −140°C and transfer into LN	In Syme's amputations group, five of six cryopreserved and replanted limbs survived 3 months	(52)
Lewis rat ovary	M2 medium containing fructose and DMSO	Programmatically cool to −140°C and induce ice nucleation at −7°C, then transfer into LN	Successful transplantation in rats of ovaries, fallopian tubes and the upper segment of the uterus en bloc after cryopreservation	(105)
Murine and porcine livers	University of Wisconsin (UW) solution supplemented with 10% (vol/vol) ethylene glycol (EG)	Directional freezing to −40°C, then transfer into −80°C freezer or LN (Rat liver); Directional freezing to −40°C (Porcine liver)	Rat and porcine livers were intact and demonstrated >80% viability	(36)
Whole sheep ovaries	University of Wisconsin (UW) solution supplemented with 10% DMSO	Directional freezing to −70°C, then transfer into LN	Three ovaries retransplanted 6 years all had intact and functional vasculature connections	(34)
Human amputated fingers	Fetal bovine serum (FBS), DMSO, RPMI 1640 medium	Programmatically cool to −80°C for 1day then transfer into LN	Cryopreserved for 81 and 5 days, respectively, both fingers were replanted successfully	(53)
Rabbit kidney	M22	Vitrification	The rabbit kidney was vitrified successfully and survived after transplantation	(66)
Rabbit jugular vein	VS55	Vitrification	Significantly improved the function of vascular tissue after cryopreservation	(106)
Human mucosal tissues	10% DMSO in fetal bovine serum	Programmatically cool to −80°C	Cryopreservation of intact cervicovaginal and colorectal tissues is effective	(107)
Human skin	FBS, DMSO, RPMI 1640 medium	Cool at 1°C/min to −80°C, then transfer into LN	Skin had good quality and high survival rate, and successfully replanted back into human body	(30)
Intact femora of *ddy* mice	DMSO	Cool at −70°C/min to −70°C for 1 day	Partially maintained the biological function of osteochondral tissue	(22)
Pancreatic islet	EG, DMSO	Induce ice nucleation at −7.5°C, cool at 0.25°C/min to −40°C then transfer into LN	Pancreatic islet cryopreservation achieved high viability, recovery, function and scalability simultaneously	(108)
Human corneas	Eusol-C preservation media	Store at −78°C	Stored for an average of 6.9 months and used for transplantation	(48)
Human adipose	Glycerol	Cool at 1°C/min to −80°C, then transfer into LN	Better structural integrity and survivability	(21)
Human brain	DMSO, glycerol	Strategically frozen to −80°C and store without rewarming	Rat supplementation experiments revealed damage to specific neuronal cells, while the synaptic networks in the hippocampus remained unaffected	(109)
Rat lung	N-(2-fluorophenyl)-D-gluconamide (2FA), DMSO	Cool at −5°C/min to −20°C	Improves alveolar cell membrane integrity and tissue structural integrity	(110)
Porcine artery	VS55 containing silica–coated iron oxide nanoparticles (sIONPs)	Vitrification and nanowarming	No significant biomechanical property changes in blood vessel length or elastic modulus compared to fresh control porcine arteries	(38)
Rat heart	VS55 containing superparamagnetic iron oxide nanoparticles (SPIONs)	Vitrification and nanowarming	No macroscopic damage is detected	(44)
Rat kidney	VS55 containing silica-coated iron oxide nanoparticles (sIONPs)	Cool at −40°C/min to −150°C achieving vitrification and nanowarming	Kidneys recovered intact without any visible cracks and showed preserved viability, architecture and intact endothelium	(45)
Rat livers	Ethylene glycol + sucrose (EG + Suc) in Euro-Collins (EC) solution	Vitrification and nanowarming	Maintained normal tissue architecture, had preserved vascular endothelium, and demonstrated hepatocyte and organ-level function	(46)

For this, scientists have used the technique of directional freezing to regulate the growth of ice crystals, where the sample moves through a temperature gradient at a constant velocity to limit the gradual growth of ice crystals across that gradient. The controlled ice crystals appear as lamellae, with cells trapped between the lamellae to reduce mechanical damage (31). Additionally, tight contact between the sample and the highly heat-conductive metal block enables efficient heat dissipation, avoiding the damage to cells caused by freeze-thaw cycles (32). Directional freezing can be used for both slow and rapid cooling, and has been extended to directional vitrification (33). Directed freezing technology demonstrates the capability to achieve uniform cooling rates in tissues and organs, and has been applied in cryopreservation experiments of various types of tissue and organs, such as ovaries (34), rat hindlimbs (35), and pig livers (36). This is a promising approach, but some research results seem to lack sufficient conviction and more efforts are still needed to advance its development.

Vitrification is the rapid freezing of a liquid to allow it to jump through the crystallization zone and form an amorphous or non-crystalline solid state (37). To achieve vitrification, cooling rates faster than the critical cooling rate (CCR) are required to prevent ice crystal formation, and it has been achieved after perfusion of small organs with CPAs (38). Similarly, a very high warming rate is required during the recovery process to avoid devitrification (39, 40). Although using CPAs can decrease both CCR and critical warming rate (CWR), the CWR is usually an order of magnitude higher than the CCR (41), and traditional convective warming is difficult to achieve such a rate. Recently reported magnetic nanoparticle-induced heating technology effectively prevents devitrification and thermal stress during the rewarming process. Ideally, organs would be uniformly loaded with CPAs and magnetic nanoparticles through optimized hypothermic machine perfusion, and then rapidly heated uniformly in a radiofrequency-induced magnetic field after vitrification. Nanowarming has been demonstrated to be feasible in the cryopreservation of various tissues and organs, such as porcine arteries, rat hearts, rat kidneys, and rat livers, although issues such as CPA toxicity, uniform perfusion and washout of the magnetic nanoparticles remain to be addressed. Nonetheless, it is undeniable that this technology has propelled the field of vitrification of organs toward new frontiers (38, 40, 42–46).

So far, the majority of tissues and organs that have been successfully cryopreserved and transplanted are small or structurally simple, such as skin (47), corneas (48), and osteochondral tissue (49). However, successful cases of complex organs are few and far between. To our knowledge, the reported successful cases include ovaries, rat hindlimbs, and human fingers. Ovarian tissue can recover its function after cryopreservation, and transplant recipients [such as sheep (50) and humans (51)] have successfully given birth to offspring. In addition, cryopreserved rat hindlimbs can survive after transplantation (35, 52), although the function of some hindlimbs may not recover. Human amputated fingers can be successfully reimplanted back into the patient's hand and restore function after 30 days of cryopreservation (53, 54). Cryopreservation of small limbs with low content of complex muscle tissue is relatively easy, but larger tissues and organs with more complex structures and functions have not yet been fully achieved, possibly because some structures are too delicate and complex to withstand the adverse effects of the cryopreservation process (53, 55). For example, the delicate capillary network of frozen kidneys can be damaged after nanowarming, with consequent impairment of organ function (56). Functional recovery after cryopreservation of vital organs such as the heart and kidneys has not been accurately assessed, as many researches fail to sufficiently assess biological characteristics and function, and *in vivo* testing is absent.

## 3. The challenges of tissue and organ cryopreservation

### 3.1. Troubles caused by ice crystals

During the cooling and rewarming processes in cryopreservation, uncontrolled ice crystal formation and growth can be fatal to tissue and organ preservation (57). For programmable slow freezing, ice crystals are difficult to avoid even with the best preservation protocols (17, 20), which can cause damage to the intercellular connection and blood vessels in tissues and organs. However, it is often difficult to achieve the optimal cryopreservation conditions for multiple cell types in an organ simultaneously (57, 58). Additionally, the phase transition process of ice crystal formation is accompanied by heat release, which can cause serious damage to tissue cells beyond a certain limit (24). It is worth noting that necessary cell-cell and cell-matrix interactions in tissues are related to intracellular ice formation (IIF), which is inseparable from the low survival rate of tissues after cryopreservation (59–61).

### 3.2. Toxicity due to high concentration of CPAs

Except for rare reports of cell CPA-free cryopreservation (62), CPAs are indispensable for both tissue and organ cryopreservation. The conventional cryoprotectant DMSO has been shown to have multiple toxic damages to cells, such as damage to proteins (63), mitochondria (64), and extracellular structures (65). Since the function of tissues and organs depends on intercellular junctions (57), damage to cell structures can result in impaired or lost function of tissues and organs after cryopreservation. Cell vitrification typically uses high concentrations of CPAs at 4–8 M, and similarly, organ cryopreservation also requires loading high concentrations of CPAs to inhibit ice crystal formation. For example, Fahy et al. (66) used a cryoprotective solution as high as 9.3 M to perfuse rabbit kidneys for vitrification. In large organs, the time of perfusing protective solution can reach several hours, and high concentrations of CPA can cause damage to cells through osmotically induced mechanical stress, and prolonged exposure can also make CPA toxicity a dominant factor in injury (4, 65). It is crucial to find a balance between the toxicity of CPA and its ability to inhibit ice formation in the cryopreservation of large tissue organs.

### 3.3. Heat and mass transfer problems in complex structures

Compared to cell suspensions, the processes of heat and mass transfer in tissues and organs appear to be much more complex. In terms of mass transfer, the loading and removal of CPAs and the distribution of water are the main considerations (67). The mass transfer process within the organ becomes complicated due to the different types and arrangements of cells, as well as the interactions between cells. This limits the diffusion of water and CPAs, resulting in difficulties achieving uniform distribution of CPAs in organs. Moreover, the excessive macroscopic volume also hinders the uniform distribution of CPAs in the surface and interior of the organ. For example, during organ perfusion, vascular cells that first come into contact with CPAs are subject to greater toxicity and injury (67–69). A similar problem to mass transfer is faced in heat transfer, which is limited by the macroscopic volume and thermal conductivity of the organ, making it difficult to achieve uniform cooling and rewarming (44, 70). As a result, organs experience thermal stress damage during cryopreservation, which often leads to the rupture of biological samples. The cracks also promote ice crystal formation, especially during the rewarming process (42, 71). The uneven distribution of CPA tends to lead to the appearance of temperature gradients and aggravates the thermal stress damage (72). The complex heat and mass transfer problems seriously hinder the tissue and organ cryopreservation, while the gold standard convective warming cannot meet the need for rapid and uniform heating of large biological samples. Urgent measures are needed to address these pressing problems.

### 3.4. Oxidative damage

Under normal physiological conditions, cells maintain a balance between oxidation and reduction reactions. However, this balance is disrupted during cryopreservation by decreased enzyme activity, ATP deficiency, and Ca^2+^ accumulation, leading to oxidative stress and the production of a large number of reactive oxygen species (ROS) (73, 74). Excessive ROS can induce a multitude of deleterious effects, including but not limited to DNA damage, protein oxidation, and lipid peroxidation (75). The adverse effects of ROS are further aggravated by the fact that mitochondria, the source of ROS, are also attacked by ROS. Damaged mitochondria produce less ATP and more ROS, which further exacerbates cellular oxidative damage (76, 77). The series of damages caused by oxidative stress can impair cellular functions and decrease vitality post-cryopreservation, accelerating cell apoptosis and necrosis in conjunction with other types of damage (78, 79). An additional problem that organs face during *ex vivo* cryopreservation is ischemia-reperfusion injury, which is caused by redox imbalance. Research has shown that a substantial amount of ROS is generated during ischemia (hypoxia) and reperfusion (reoxygenation) (80, 81), leading to tissue damage and compromising the quality of the transplantable organs.

## 4. Possible research directions for tissue and organ cryopreservation

### 4.1. Reducing the toxicity caused by CPAs

The toxicity of conventional CPAs has been a persistent obstacle to the development of cryopreservation, prompting researchers to explore solutions. Substantial research has been devoted to the development of high-efficiency and low-toxicity CPAs, such as antifreeze proteins (82), macromolecular polymers (83, 84), and nanomaterials (85–87). Besides, combining different CPAs in specific molar ratios as a CPA cocktail can reduce toxicity. Examples of such cocktails include improved VS41A, Natural Deep Eutectic Systems (NADES), and others used for vitrification of biological samples (88–91). Notably, mathematical models have been used in the development of low-toxicity vitrification solutions (66, 92, 93), a form similar to computer simulation of drug development or a trend in the development of new CPAs. And the method of hypothermic machine perfusion with multiple steps of loading/unloading CPAs can reduce the toxicity and osmotic damage to organs (94). In vitrification, toxicity damage may have become the main challenge (45), and identifying more favorable perfusion schemes and ideal CPAs will strongly promote the development of cryopreservation.

### 4.2. Finding new methods of cryopreservation

Tissue and organ cryopreservation is a huge challenge that requires cross-disciplinary participation and more effective new technologies to break existing barriers. According to recent studies, strategies such as nanoparticle-mediated intracellular delivery of trehalose (95), microfluidics-controlled hydrogel for cell encapsulation (96), and sand-mediated ice nucleation (97) have achieved good results in cell cryopreservation. Physical fields have also played a huge role in cryopreservation, such as magnetic fields (45) and laser fields (98), which can mediate rapid nanowarming to avoid ice crystals during the thawing process. And the appropriate use of additives has been found to improve the recovery of cells after cryopreservation, such as antioxidants and caspase inhibitors (99–101). In addition, the biochemical perspective has given researchers much insight. Dou et al. significantly enhanced cold tolerance by feeding L-proline to Japanese carpenter ants, innovatively linking genetic variation to cold resistance in ants through exogenous feeding of CPA (102). This indicates that a bionic strategy of learning from nature can provide new techniques and ideas for tissue and organ cryopreservation.

### 4.3. Exploring the mechanism of damage in cryopreservation

The current understanding of the damage mechanisms during cryopreservation mainly focuses on the dispersed cellular level, while the mechanisms targeting the damage to the multi-level structure and physiological functions of tissues and organs are still unclear. Research on the cryopreservation of tissues and organs mainly focuses on the integrity of macroscopic structures, and there is a lack of in-depth exploration at the cellular and molecular levels within the organs. For example, recent research on vitrification and nanowarming of rat hearts showed that the macroscopic structure remained intact, but the cause of functional damage was not clear and it was speculated that it may be due to the toxicity of the CPA or perfusion injury (103). However, the toxic mechanism of the vitrification solution on tissue organs has not been elucidated, and the mechanism of toxic damage still needs to be demonstrated at the microscopic molecular level (65, 88). Without further explanation of the damage mechanism of tissue and organ during cryopreservation, it will be difficult to apply targeted methods to improve the effectiveness of cryoprotection.

## 5. Conclusion

Cryopreservation of a small number of tissues and organs has been clinically adopted, but complex organs like the heart may fail to recover due to damage to their highly refined structure. During cryopreservation, tissues and organs are primarily subjected to damage from ice crystal formation, thermal stress, reactive oxygen species, and toxicity of CPAs, and the biological features and functional testing of the organs after recovery are relatively lacking. The combination of vitrification and nanowarming technology has shown remarkable performance in maintaining the structural integrity of small animal organs, and expanding this technique to larger tissue and organs with *in vivo* functional tests after recovery is a promising future direction. In-depth exploration at the cellular and molecular levels within organs is beneficial for elucidating the specific mechanisms of damage in cryopreservation, thereby providing new research perspectives. Optimizing the vitrification solution and machine perfusion protocols can reduce toxic damage during cryopreservation, while searching new efficient and low-toxic CPAs remains an important means of solving this problem. Furthermore, interdisciplinary new technologies are an important direction for future development, which may bring unexpected breakthroughs in improving the quality of tissue and organ preservation.

## Author contributions

JC has made substantial contributions to the conception and design of this work. XL, YH, and XC have took part in revising work critically for important intellectual content. ST has revised work and approved the final version to be published. All authors contributed significantly to the writing of the manuscript.
