# Supplementary material for: Cryopreservation of tissues and organs: present, bottlenecks, and future
Source: Front Vet Sci. 2023 May 25;10:1201794. doi: 10.3389/fvets.2023.1201794 (PMC10248239; doi:10.3389/fvets.2023.1201794)
Supplement: Supplementary file 1 [file Table_1.DOCX]

Supplementary Material

# Supplementary Table

Table S1. Comparison of the advantages and disadvantages for the various cryopreservation methodologies.

| Processes of cryopreservation | Advantages | Disadvantages | Ref |
| --- | --- | --- | --- |
| Programmable slow freezing | - Conventional equipment - More mature technology - Low concentration of CPAs | - Damage from ice crystals - Typically applied to small-sized tissues and organs | (Hovatta, 2005; Campbell et al., 2014; Wang et al., 2019; Bojic et al., 2021; Criswell et al., 2023) |
| Directional freezing | - Regulating the growth of ice crystals by controlling the thermal gradient - Efficient heat dissipation reduces freeze-thaw cycle damage - Suitable for slow and rapid freezing | - Require professional equipment - Still facing damage caused by ice crystals | (Gavish et al., 2008; Arav and Natan, 2012; Maffei et al., 2013; Arav et al., 2017; Arav, 2022) |
| Vitrification and nanowarming | - Ice-free cryopreservation - Expansion to larger volumes of tissue and organ - Reduce thermal stress damage and avoid devitrification | - Toxicity of high concentrations of CPAs - Uneven distribution and residual unload of nanoparticles - Necessitate rapid cooling and warming rates - Require high level of operational skills | (Fahy et al., 2004; Manuchehrabadi et al., 2017; Finger and Bischof, 2018; Sharma et al., 2021; Gao et al., 2022; Sharma et al., 2023) |

# Reference

Arav, A. (2022). Cryopreservation by Directional Freezing and Vitrification Focusing on Large Tissues and Organs. *Cells* 11(7). doi: 10.3390/cells11071072.

Arav, A., Friedman, O., Natan, Y., Gur, E., and Shani, N. (2017). Rat Hindlimb Cryopreservation and Transplantation: A Step Toward "Organ Banking". *American Journal of Transplantation* 17(11)**,** 2820-2828. doi: 10.1111/ajt.14320.

Arav, A., and Natan, D. (2012). Directional freezing of reproductive cells and organs. *Reprod Domest Anim* 47 Suppl 4**,** 193-196. doi: 10.1111/j.1439-0531.2012.02075.x.

Bojic, S., Murray, A., Bentley, B.L., Spindler, R., Pawlik, P., Cordeiro, J.L., et al. (2021). Winter is coming: the future of cryopreservation. *BMC Biol* 19(1)**,** 56. doi: 10.1186/s12915-021-00976-8.

Campbell, B.K., Hernandez-Medrano, J., Onions, V., Pincott-Allen, C., Aljaser, F., Fisher, J., et al. (2014). Restoration of ovarian function and natural fertility following the cryopreservation and autotransplantation of whole adult sheep ovaries. *Hum Reprod* 29(8)**,** 1749-1763. doi: 10.1093/humrep/deu144.

Criswell, T., Swart, C., Stoudemire, J., Brockbank, K.G.M., Powell-Palm, M., Stilwell, R., et al. (2023). Freezing Biological Time: A Modern Perspective on Organ Preservation. *Stem Cells Transl Med* 12(1)**,** 17-25. doi: 10.1093/stcltm/szac083.

Fahy, G.M., Wowk, B., Wu, J., Phan, J., Rasch, C., Chang, A., et al. (2004). Cryopreservation of organs by vitrification: perspectives and recent advances. *Cryobiology* 48(2)**,** 157-178. doi: 10.1016/j.cryobiol.2004.02.002.

Finger, E.B., and Bischof, J.C. (2018). Cryopreservation by vitrification: a promising approach for transplant organ banking. *Curr Opin Organ Transplant* 23(3)**,** 353-360. doi: 10.1097/MOT.0000000000000534.

Gao, Z., Namsrai, B., Han, Z., Joshi, P., Rao, J.S., Ravikumar, V., et al. (2022). Vitrification and Rewarming of Magnetic Nanoparticle-Loaded Rat Hearts. *Adv Mater Technol* 7(3). doi: 10.1002/admt.202100873.

Gavish, Z., Ben-Haim, M., and Arav, A. (2008). Cryopreservation of whole murine and porcine livers. *Rejuvenation Res* 11(4)**,** 765-772. doi: 10.1089/rej.2008.0706.

Hovatta, O. (2005). Methods for cryopreservation of human ovarian tissue. *Reprod Biomed Online* 10(6)**,** 729-734. doi: 10.1016/s1472-6483(10)61116-9.

Maffei, S., Hanenberg, M., Pennarossa, G., Silva, J.R., Brevini, T.A., Arav, A., et al. (2013). Direct comparative analysis of conventional and directional freezing for the cryopreservation of whole ovaries. *Fertil Steril* 100(4)**,** 1122-1131. doi: 10.1016/j.fertnstert.2013.06.003.

Manuchehrabadi, N., Gao, Z., Zhang, J., Ring, H.L., Shao, Q., Liu, F., et al. (2017). Improved tissue cryopreservation using inductive heating of magnetic nanoparticles. *Sci Transl Med* 9(379). doi: 10.1126/scitranslmed.aah4586.

Sharma, A., Lee, C.Y., Namsrai, B.E., Han, Z., Tobolt, D., Rao, J.S., et al. (2023). Cryopreservation of Whole Rat Livers by Vitrification and Nanowarming. *Ann Biomed Eng* 51(3)**,** 566-577. doi: 10.1007/s10439-022-03064-2.

Sharma, A., Rao, J.S., Han, Z., Gangwar, L., Namsrai, B., Gao, Z., et al. (2021). Vitrification and Nanowarming of Kidneys. *Adv Sci (Weinh)* 8(19)**,** e2101691. doi: 10.1002/advs.202101691.

Wang, Z.T., Zhu, L., Kou, W., Sun, W.H., He, B., Wang, C.X., et al. (2019). Replantation of Cryopreserved Fingers: An "Organ Banking" Breakthrough. *Plastic and Reconstructive Surgery* 144(3)**,** 679-683. doi: 10.1097/Prs.0000000000005979.
